# Detection of mast cells in ameloblastomas and odontogenic keratocysts

**DOI:** 10.4317/jced.56723

**Published:** 2020-08-01

**Authors:** Erison-Santana dos Santos, Richard-Ribeiro-Alonso de Andrade, Gerhilde-Callou Sampaio, Raisa-Queiroz Catunda, Emanuel-Sávio-de Souza Andrade

**Affiliations:** 1 DDS, MSc Student, Department of Oral Diagnosis, Piracicaba Dental School, University of Campinas, Piracicaba, São Paulo, Brazil; 2 DDS, MSc, Assistant Professor, Department of Surgery and Oral Pathology, Pernambuco Dental School, University of Pernambuco, Camaragibe, Pernambuco, Brazil; 3 DDS, MSc, PhD, Adjunct Professor, Department of Surgery and Oral Pathology, Pernambuco Dental School, University of Pernambuco, Camaragibe, Pernambuco, Brazil; 4 DDS, MSc, PhD Candidate, Department of Medicine and Dentistry, School of Dentistry, University of Alberta – Edmonton, AB – Canada; 5 DDS, MSc, PhD, Full Professor, Department of Surgery and Oral Pathology, Pernambuco Dental School, University of Pernambuco, Camaragibe, Pernambuco, Brazil

## Abstract

**Background:**

MCs (MCs) have been ascribed to mediating several diseases, including malignant neoplasms. These cells can play a role in angiogenesis, tissue remodeling and immune modulation and favor neoplasm progression. Despite the studies analyzing the contribution of MCs in odontogenic lesions, its biological behavior in ameloblastomas (AMBs) and odontogenic keratocysts (OKCs) remains unclear. This study aims to detect MCs in OKCs and AMBs and clarify the role of MCs in these lesions.

**Material and Methods:**

A total of 40 odontogenic lesions were analyzed. This included 20 OKCs and 20 AMBs, 10 being the solid type and the other 10 being the unicystic type of AMB. All cases were histologically reviewed in hematoxylin-eosin. Clinical data, such as age, gender, location, size, radiographic presentation and, histologic patterns were collected from the clinical charts. The Mann–Whitney U test (MWU) was used verify the hypothesis, through inferential statistics. The level of significance used in the statistical test was 0.5%.

**Results:**

MCs were observed in 60% of OKCs, and 35% of AMBs. The ratio of MCs observed in OKCs was 0.37, 0.48 in solid AMBs and 0.01 in unicystic AMBs. There was no significant difference between number of MCs in AMBs and OKCs, however, a significant difference was observed between solid and unicystic AMBs (*p* ≤ 0.01).

**Conclusions:**

MCs may play an important role in the biological behavior of AMBs and OKCs. However, in this study it was not possible to confirm the contribution of MCs in the biological behavior of these lesions and more studies are needed to clarify this relation.

** Key words:**AMB, OKC, MCs, histochemistry, toluidine blue.

## Introduction

Odontogenic lesions are derived from ectomesenchymal and/or epithelial tissues of either the dental organ or its associated structures (1). In the context of these lesions, AMBs and OKC are particularly important because of their intriguing biological behavior (1). OKCs are lesions found in adults, mainly men, in the posterior region of the jaw (2,3). Normally, they are asymptomatic, but large cysts may be symptomatic (2,3). AMBs are significant due to their frequency, aggressiveness and high recurrence rates (2,3). AMBs have three variants: conventional, unicystic and peripheric. They are frequently found in the posterior region of the jaw, asymptomatic, slow-growing pattern but with locally invasive behavior (4). Radiographically, they can appear as unilocular or multilocular radiolucent lesions (2,3,4). Despite the studies utilizing advanced techniques of molecular biology to comprehend the etiopathogenesis of these lesions, its molecular mechanism remains unclear.

However, recent studies have showed that MCs may be involved in progression of pathologies such as odontogenic cysts and tumors (2,3), including OKCs (4) and AMBs (5). Since its discovery by Paul Ehrlich in 1879, MCs have been the subject of several studies to clarify their role in immunological responses (5,6). First studies about these cells were based on morphological features to identify its distribution in pathological and physiological conditions (6,7). The origin of MCs is complex and poorly explained, although probably they arise from the bone marrow (7). They migrate into the tissue and assume their typical granular morphology, defending the host against pathogens (5-7). Moreover, some studies show that MCs may participate in lesion progression through angiogenesis, tissue remodeling and immune modulation (8,9), including odontogenic lesions (2,3). Analysis of MCs expression has been used to identify and study the role of these cells in different odontogenic lesions (1-3). Diverse methods have been used to study these cells such as staining with toluidine blue by histochemistry and immunohistochemistry analysis of proteins called endopeptidases as chymases and trypsin (1-3).

Although there are some studies analyzing the role of MCs in odontogenetic lesions, none of them compare the presence of MCs in AMBs and OKCs. These lesions deserve careful attention due to their clinical aggressive behavior, high rates of recurrence and, for AMB, its capacity to metastasize to distant sites. Therefore, the purpose of this study was to analyze and compare the presence of MCs between OKCs and AMBs to understand the influence of MCs in biological behavior of these odontogenic lesions. The data from this analysis would provide a better understanding of the role of these cells and how AMBs and KCOs develop and are maintained.

## Material and Methods

-Study design and ethical considerations 

The present study consisted of 40 previously diagnosed odontogenic lesions embedded in paraffin. This included 20 OKCs and 20 AMBs, 10 being the solid type and the other 10 being the unicystic type of AMB. This study was approved by the Ethics Committee of the University of Pernambuco (protocols CAAE 04314712.8.0000.5207 / CAAE 11365412.2.0000.5207). Following the recommendations of the Helsinki Declaration, the anonymity of participants was assured.

-Toluidine blue stained sections

Firstly, these cases were retrieved from archives of pathological anatomy laboratory from School of Dentistry of Pernambuco, University of Pernambuco, Camaragibe, Brazil. Slides were analyzed by an experienced pathologist to confirm diagnosis based on the classification of tumors and odontogenic cysts of the World Health Organization (7). After confirmation, paraffin-embedded blocks were analysed, 5μm sections were prepared and stained with the standard toluidine blue method (0.1%) to investigate for the presence of MCs.

The specimens were deparaffinized in xylene (twice for 7 min) and 80% absolute alcohol (5 min) and 90% (5 min) at room temperature. Then they were immersed in distilled water for 5 minutes, toluidine blue solution for 3 minutes and placed in running water for 5 minutes. After staining, they were placed in the oven at 37º C for 24 hours, immerse in xylene for assembly and then mounted in permount.

-MCs counting

The counting of stained cells was performed under a light microscope (Olympus CH30RF200) at a 400x magnification in 10 consecutive fields on each slide in the full extension of the lesions. The selection of the field for cell counting was performed with the aid of ocular lens containing a 4-quadrant gradient, with a total area of 10x10 mm (100mm2) and, in each quadrant, an area of 5x5 mm (25mm2). In order to avoid repetition of the cell count in the same quadrant and considering that MCs stained were in the subepithelial region of the cystic lesions and around the ameloblastic epithelium in the solid lesions. The protocol was to count the cells at the upper left quadrant of the gradient, along the subepithelial portion of each lesion.

-Statistical analysis

Statistical analysis was carried out with the aim of comparing mast cell counting using the Kolmogorov–Smirnov test to evaluate normality of the data. The Mann–Whitney U test (MWU) was used verify the hypothesis that the correlation is null, through inferential statistics. The level of significance used in the statistical tests was 0.5%. The data was entered in the Excel spreadsheet and the software used to obtain the statistical calculations was the Statistical Package for Social Sciences version 20.0 (SPSS, Chicago, IL).

## Results

**Figure 1:**
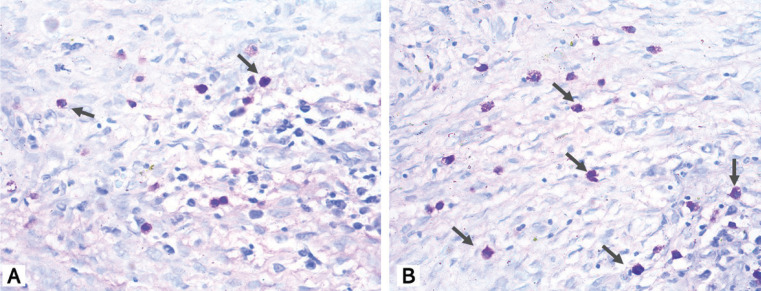
Morphologic aspect of mast cells exhibiting a rounded or elongated shape, with granular appearance and blue, purple and violet coloration.

**Figure 2:**
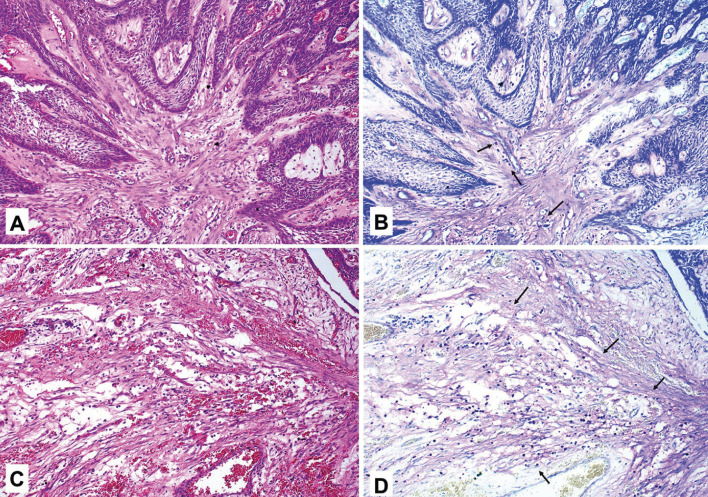
Amount of mast cells near the islands or ameloblastic epithelium strands in solid ameloblastomas.

**Figure 3:**
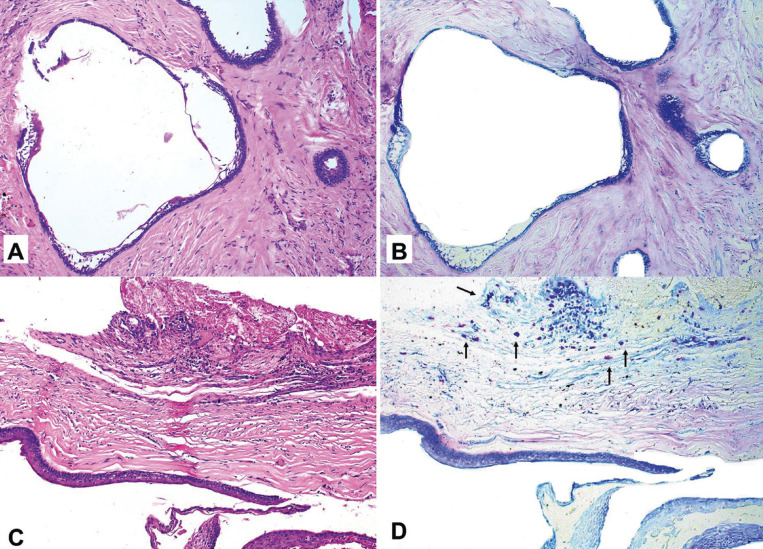
A and B Unicystic ameloblastoma (Luminal). There are no mast cells observed in these lesions. C and D The presence of mast cells in the subepithelial region (odontogenic keratocyst).

Morphologically MCs exhibited a rounded or elongated shape, with granular appearance and blue, purple and violet coloration (metachromasia) (Fig. 1). MCs are present in a total 47.5% of analyzed lesions. A slight greater amount was observed in AMBs than in OKCs, however, there was not any significant difference in mast cell count between these lesions (*p-value*s > 0.05, MWU). MCs were found near the islands or ameloblastic epithelium strands in solid lesions (Fig. 2), although they were also seen in other regions. In OKCs, they were found in the subepithelial region (Fig. 3). There was only one case of unicyst AMB that presented MCs (Fig. 3).

Moreover, regarding the clinical profile of patients studied, there was no significant difference in the clinical information (Table 1). However, there was a significant difference between the presence of MCs and radiographic and histologic patterns (*p*-values = 0.037 and 0.000, respectively, MWU) (Table 2). MCs were found in 12 (60%) OKCs and 7 (35%) AMBs, of which 1 (10%) was unicystic and 6 (60%) were solid AMBs. The average number of MCs found in all lesions was 0.37 in OKCs and 0.24 in AMBs. For AMBs the average was 0.48 in solid and 0.01 in unicystic AMBs (*p* ≤ 0.01, MWU). The sample with the highest number of MCs was identified in the OKC sample having a total of 17 MCs. A summary of the mean distribution of MCs per slide can be found in Figure 3.

**Table 1:**
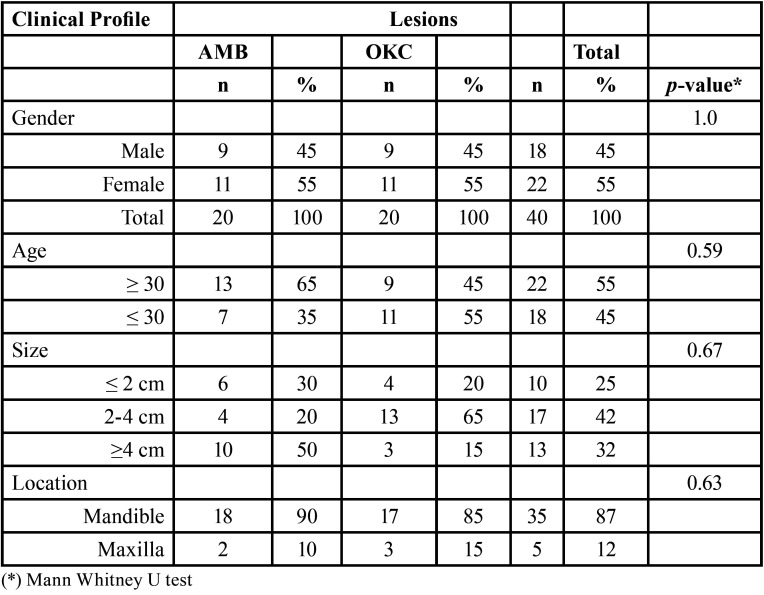
Clinical profile of ameloblastoma and odontogenic keratocystic.

**Table 2:**
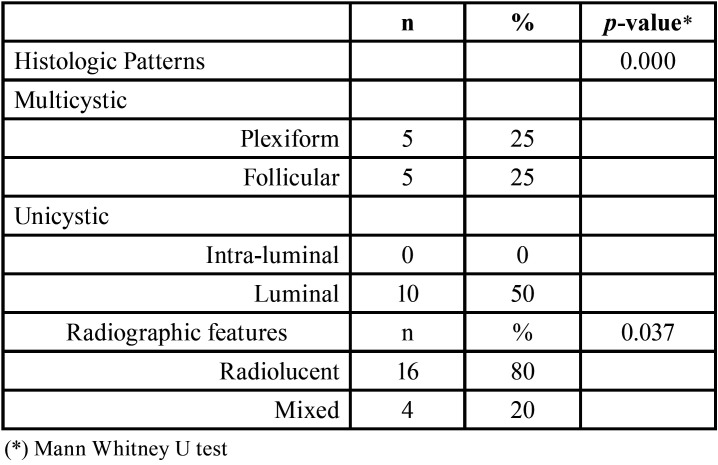
Histologic patterns and radiographic features of ameloblastoma.

## Discussion

Odontogenic cysts and tumors are relatively frequent lesions in the oral cavity, affecting patients of different age groups (6). The etiology of these lesions is still uncertain, but they seem to be related to the remnants of the dental lamina and the odontogenic epithelium (4). Genetic alterations may play a role in the etiology and have been associated with the high aggressiveness of OKCs, (6,8,9).

These lesions present clinically with varying measurements, with or without symptoms, size dependant. Unlike AMB, OKCs do not exhibit bone expansion with well-defined borders (4). AMBs on the other hand can be uni or multilocular radiographically, with irregular borders (4,10). Histopathologically, AMBs present with a variable pattern having a predominance of ameloblastic epithelium islands in the solid type and the same type of epithelium invading the lumen of the cyst, in the case of the unicystic lesions (1). The OKC presents as a thin capsule lesion with few cell layers (8,4). The basal layer presents in a palisade with the surface between the epithelium and the underlying connective tissue (4,6,8).

There are some studies showing the relation of the MCs with several diseases, including malignances (6,7). In odontogenic lesions, such as cysts and tumors, the role of MCs is uncertain still. However, because of its location and distribution in odontogenic cysts, including OKCs, they have been associated with the aggressiveness of these lesions (1-3,9). In odontogenic tumors, the density of MCs and micro vessels were greater in AMBs (an aggressive lesion) than in adenomatoid odontogenic tumors (non-aggressive lesion), which could be a potential indicator that MCs participate in the progression of tumors through angiogenesis (11). The tumor microenvironment is complex, with a number of stromal components such as cells and vessels, that can interact and play a role by in tumor growth (11). MCs play an important role in the formation of new micro vessels by angiogenesis (6,7). They produce and release numerous factors such as heparin, vascular endothelial growth factor, platelet-activating factor, tryptase, chemotactic mediators, and fibroblast growth factor that play pivotal function in angiogenesis (6,7). It was suggested that the formation of new vessels near to lesions may favor the tumor growth (10,12-14). However, in odontogenic cyst studies show that there is no significative association between numbers of MCs and micro vessels (10,12-14). Despite these findings, we believe that clinical and molecular behavior of these lesions are associated with other several factors and isolated events are insufficient to lead to the development and progression of cysts and tumors.

In the present study, toluidine blue was used to stain MCs and to investigate the possible contribution to the biological behavior of these cysts and tumors. There was no statistically significant difference between presence of MCs in AMBs and OKCs. In the AMBs group, the solid presented with a greater number of MCs then the unicystic (*p* ≤ 0.01). Unicystic AMBs are less aggressive and with low rates of recurrence than solid and OKCs. This lends credence to the possible role of MCs in more hostile biological behavior (15). However, a previous study demonstrated that there is no significant difference in the density of MCs in both solid and unicystic AMBs even when different areas of lesions were considered (1). But, interestingly, these cells are more frequent in solid AMB than adenomatoid odontogenic tumor which are considered less aggressive. They were also more frequent in syndromic OKC than in non-syndromic (1). To best of our knowledge, syndromic lesions are more commonly related to higher aggressiveness and bone destruction when compared with non-syndromic lesions (10,16). These findings are probably due to different techniques utilized in staining of lesions and sample size on each study.

Furthermore, it has been suggested that MCs degranulation products may participate in the progression of cystic lesions due to the destruction of the extracellular matrix, stimulation of cytokine production and then, facilitating bone resorption and lesions expansion (1,2,11,16). Moreover, it was demonstrated that MCs in connective tissue are associated with cyst expansion through secretion of chemical mediators such heparin and hydrolytic enzymes (1,11). Production and release of heparin and TNF-α cause osteolytic activity, consequently, the bone surrounding cystic lesions is destroyed causing more expansion (1,11). In our study, the overall presence of MCs observed in OKCs was low due to staining method utilized to visualize these cells. In a preview study, a significant number of MCs was found near blood vessels of OKCs (2). In contrast, a study evaluated MCs in odontogenic radicular keratocysts (RC), and pericoronal follicles (PF) and it was observed that these cells were present in lower number in OKCs compared with RCs, but they were substantially higher when compared to PFs (13). The authors suggest that these differences are intrinsically due to the inflammatory nature of these lesions, even though a number of MCs were found in non-inflamed OKCs (13). Few blood vessels were observed in our study; therefore, according to our results, it is difficult to understand the relation of proximity of MCs and blood vessels with the biological behavior of these lesions. More studies with accurate methods are needed to clarify this relationship.

Besides, a significant difference was found between histologic patterns and the presence of MCs in AMBs. In a database review at a single institution, 54 patients having a histopathological diagnosis of AMB were examined following treatment for recurrent lesions. It was observed that the follicular type of AMB had a higher risk of recurrence. In contrast, the unicystic type and demoplastic type of AMBs had a lower risk of recurrence (17). According to our results, it is not possible to conclude that there is a relationship between the presence of MCs and recurrence rates due the small sample size analyzed in this study and insufficient information about patients’ follow-up.

The behavior of odontogenic cysts and tumors appears to be complex and depends on a combination of biological events. To the best of our knowledge, the progression of these lesions may not be related only to a specific event, but to the combination of several external factors inherent to the injury itself (1-3). Among these factors, it is possible to suggest the presence of MCs as a promoter of progression and aggressiveness, as suggested by some authors (1-3,9,11-13). Despite these studies, more robust studies are needed to define the roles of MCs in the progression of lesions, including odontogenic lesions.

Based on our study, it was observed that the presence of MCs was a slight greater in samples of AMBs than in OKCs, and in a higher number in solid than in unicystic AMBs. Although the role of MCs in these lesions remains undefined, the presence of these cells in more aggressive lesions can provide an important insight on tumor progression (16,18). Therefore, according to our results and previous studies, it was suggested that MCs may be involved with aggressiveness of odontogenic tumors due to the significant presence in solid AMBs, more aggressive clinical lesions with high rates of recurrence (4,17). On the other hand, it was not possible associate the presence of these cells with behavior of odontogenic keratocytes or the role of micro vessels in both lesions. Further studies with more sophisticated techniques are needed to confirm and to understand the contribution of MCs in the biological behavior of odontogenic cysts and tumors.

It is important to highlight some limitations present in this study such as size of sample. Moreover, we utilized only histochemistry techniques to identify MCs, but it is interesting to perform an immunohistochemical panel to better elucidate the presence and distribution of these cells across the connective tissue. Unfortunately, this study was performed in a center of oral diagnosis and important clinical data about these patients are not available to correlate the presence of MCs with recurrence rates.

## Conclusions

MCs play important roles in biological functions. Their participation in the biological behavior of AMBs and OKCs remain unclear, although evidences indicate that these cells may play important role in tumor progression and maintenance. In this study, it is not possible conclude that MCs can influence the clinical and molecular behavior of AMBs and OKC. Therefore, more studies are necessary to elucidate the association of these cells in these lesions.
